# Growing, Structure and Optical Properties of LiNbO_3_:B Crystals, a Material for Laser Radiation Transformation

**DOI:** 10.3390/ma16020732

**Published:** 2023-01-11

**Authors:** Mikhail Palatnikov, Nikolay Sidorov, Alexandra Kadetova, Roman Titov, Irina Biryukova, Olga Makarova, Diana Manukovskaya, Natalya Teplyakova, Ilja Efremov

**Affiliations:** Tananaev Institute of Chemistry-Subdivision of the Federal Research Centre «Kola Science Centre of the Russian Academy of Sciences», 184209 Apatity City, Russia

**Keywords:** charge, crystal, lithium niobate, doping, boron, traces, full-profile XRD analysis

## Abstract

Physical and chemical properties have been studied in lithium niobate (LiNbO_3_, LN) crystals grown by Czochralski from a boron doped melt. Optical uniformity and optical damage resistance of LiNbO_3_:B crystals have been compared with control crystals of nominally pure congruent (CLN) and near-stoichiometric (NSLN K_2_O) composition. LiNbO_3_:B crystals structure has been studied. Studied LiNbO_3_:B crystals have been grown from differently synthesized charges. The charges have been synthesized from a mixture Nb_2_O_5_:B-Li_2_CO_3_ using homogeneously doped Nb_2_O_5_:B precursor (sample 1, (B) = 0.0034 wt% in the charge) and by a direct solid phase synthesis from Nb_2_O_5_-Li_2_CO_3_-H_3_BO_3_ mixture (sample 2, (B) = 0.0079 wt% in the charge). Only traces of boron (10^−5^–10^−4^ wt%) have been detected in the samples. We have established that concentration of anti-site defects Nb_Li_ is lower in both LiNbO_3_:B than in CLN crystals. XRD analysis has confirmed that B^3+^ cations localize in faces of tetrahedral voids O_4_ of LN structure. The voids act as buffers at the anion sublattice distortion. Sample 1 has been shown to have a structure closer to NSLN K_2_O crystal than sample 2. We have also shown that the chemical purity of LN crystal increases compared to the melt purity because boron creates strong compounds with impurities in the melt system Li_2_O-Nb_2_O_5_-B_2_O_3_. Metals impurities thus stay in the melt and do not transfer to the crystal.

## 1. Introduction

One of the most important optically nonlinear materials is lithium niobate (LiNbO_3_, LN) single crystal. It has a variable phase composition with a wide homogeneity area on a phase diagram (44.5–50.5 mol% Li_2_O) [1,2]. LN physical characteristics are changed by stoichiometry (R = (Li)/(Nb) value) variation and doping with metals [1,2,3]. Dopants should have charge between Li^+^ and Nb^5+^ [4]. Metal cations localize in octahedral (O_6_) voids of the crystals structure. Their incorporation usually changes alteration of intrinsic cations along the polar axis and distorts oxygen frame. The frame distortions are mainly caused by changes in O-O and Me-O (Me-Li, Nb, dopant, impurity) bond lengths. The spontaneous polarization determines the ferroelectric, and the polarizability of the MeO_6_ clusters determines the nonlinear optical properties of the LN crystal [4]. They both change during doping. Doping by high concentrations of non-photorefractive metal cations (Mg, Zn, Gd, In, Sc…) is a sufficiently effective way to decrease photorefractive effect in LN crystals. However, this often leads to formation of complex structural defects and compositional non-uniformity of a LN crystal. Nominally pure congruent (CLN) crystals are highly compositionally and optically perfect, yet they have a high photorefractive effect. The refractive index in the bulk of the stoichiometric crystal, on the contrary, is usually highly non-uniform. Stoichiometric crystals usually have high photorefractive sensitivity.

Chemical bonds mechanisms of non-metal cations are different from those of metal cations. As a result, mechanisms of non-metal cations influence on physical and chemical characteristics of a crystal-melt system are different; they are unable to incorporate into the oxygen octahedra O_6_ of such compounds as Nb_2_O_5_ or LiNbO_3_. This is why studies on LN doping by non-metal cations are so scarce. However, oxides of chemically active non-metals can be used as a flux during crystals growth. In this case, the elements can change the physical and chemical characteristics and structure of the melt. This will unambiguously change structure and chemical properties of LN crystals [5,6,7,8]. The Curie temperature of boron doped LN crystals is 40 K higher than that of CLN [9,10,11]. This indirectly indicates that the Li/Nb ratio of LiNbO_3_:B crystal is close to the corresponding ratio of stoichiometric LN crystal. The Li/Nb ratio in the melt of boron doped crystals corresponded to a congruent LN crystal composition, which is curious. Optical characteristics of LiNbO_3_:B crystals also change, both photovoltaic and diffusion fields increase, while the photorefractive effect decreases [9,12]. 

Mass spectrometry has determined that the boron concentration in all studied LiNbO_3_:B crystals is extremely low, 10^−5^–10^−4^ wt% [9]. This means that boron hardly incorporates into the LiNbO_3_:B crystals. Such a concentration is at a level of metal impurities traces; impurities inevitably exist in the charge and, as a result, in octahedral voids of even pure LN crystal structure. 

It is quite difficult to determine the light element boron by XRD in the LN structure in such small amounts. This is why only model calculations can be applied in this case [9,13]. The model was calculated on the base of a LN structure fragment (a cluster). The cluster consisted of 2 Li^+^ cations, two Nb^5+^ cations, one B^3+^ cation and twenty O^2−^ anions [9,13]. The cluster included six oxygen octahedra: 2 LiO_6_, 2 NbO_6_ and 2 VO_6_, where V is a vacancy. The cluster also contained two tetrahedral voids O_4_, which are formed by octahedra in the cluster. The main goal of the model calculations was determination of how B^3+^ cation interacts with the surrounding LN structure fragment in dependence of its position against tetrahedral voids faces [9,13].

**Figure 1:**
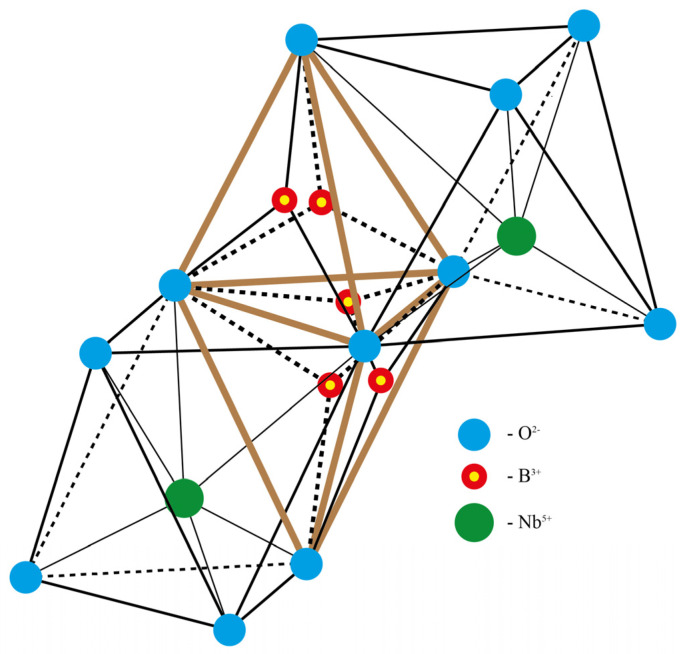
Most possible locations of B^3+^ cation as a part of [BO_3_]^3−^ group: in tetrahedra faces formed by lithium or vacant O_6_ octahedra; in a plane separating oxygen-tetrahedral layers.

Model calculations [9,13] demonstrate that B^3+^ cations having a very small radius (~0.15 Å) localize not in octahedral but in tetrahedral voids as a part of [BO_3_]^3−^ groups, Figure 1. Calculations have revealed that localization of the B^3+^ cation in a tetrahedra face formed by niobium octahedra is energetically disadvantageous. At the same time, the sum of the Coulomb interaction energies is much lower when the B^3+^ cation is located in a tetrahedra face formed by other (lithium or vacant) octahedra of LN structure. The same concerns B^3+^ cations located in a plane separating oxygen-tetrahedral layers [9,13]. The calculation results determine all possible locations of B^3+^ cations location in the LN structure, Figure 1.

A significant distortion of oxygen-octahedral clusters, MeO_6,_ was registered during analysis of Raman spectra in the area of oxygen octahedra vibrations (550–900 cm^−1^) and IR absorption spectra in the area of OH groups stretching vibrations (3420–3550 cm^−1^) [9,12,14]. These experimental data indirectly indicate changes in O-O and O-Me bonds length and probably changes in concentration and type of point defects of the cation sublattice of LN crystals at their doping by boron. Changes in O-O and Me-O bonds lengths and in point defects localization is an indirect proof of localization of boron traces in tetrahedral voids O_4_. Such data can be obtained by a full profile X-ray structure analysis. The analysis provides reliable information on the coordinates of atoms and their spatial arrangement in the crystal lattice. 

The present work considers physical–chemical particularities of growing LN crystals from boron doped melts obtained from a charge prepared due to different technologies. Optical and structural characteristics of LiNbO_3_:B crystals are compared with those of control crystals, congruent (CLN) and near-stoichiometric NSLN K_2_O (near-stoichiometric LN crystal grown from a congruent melts with alkali flux K_2_O). The structure of nominally pure LN crystals with a different R = (Li)/(Nb) ratio was studied previously in detail in a number of papers [15,16,17,18,19]. According to [15,16,17,18,19], the unit cell of ferroelectric LN phase has a space group C^6^_3V_ (R3c). The structure is based on a slightly deformed oxygen octahedra linked through edges and faces. The oxygen framework of the lattice is built according to the densest hexagonal packing. The octahedral voids of the framework, located along the polar axis, are only two-thirds occupied by intrinsic (Li, Nb), doping and impurity cations. The other third of the octahedra is empty. Two different metal–oxygen bonds can be found in Li^+^ and Nb^5+^ octahedra. This provides a large anisotropy of the crystal field in the direction of the polar axis. Nb-O distances are 2.112 and 1.889 Å, Li-O distances are 2.068 and 2.238 Å; while the sum of ionic radii of Li and O ions is 2.00 Å, and of Nb and O ions, 2.02 Å. This means that the lithium cation is located in its octahedron almost freely, and the niobium cation is rigidly fixed by a covalent bond.

The new goals of this work (actually the novelty) not achieved in our previous studies of LiNbO_3_:B crystals are the following:

- To prove experimentally the similarity of the LiNbO_3_:B crystals structure and composition to that of SLN (stoichiometric LN crystal). Previously, only assumptions about this were made; assumptions were confirmed only by indirect facts: a change in the Curie temperature, Raman spectra, etc.
- To prove the possibility of increasing the chemical purity of LiNbO_3_:B crystals during melt “purification” in the Li_2_O-Nb_2_O_5_-B_2_O_3_ system. Boron forms strong compounds with impurity metal cations in the melt. Impurities are thus removed from the crystallization process. To show that the chemical purity of LiNbO_3_:B crystals is higher than the purity of the initial charge, this is confirmed experimentally. To justify the possibility by thermodynamic calculations.
- To consider the physicochemical characteristics expressed by the phase diagram of the Li_2_O-Nb_2_O_5_-B_2_O_3_ system. To obtain the features of crystallization of a boron-containing melt of LN from this phase diagram. To find that such a melt makes it possible to obtain concentrationally and optically uniform LiNbO_3_:B crystals. To show that the production of LiNbO_3_:B crystals of high optical quality is determined by the boron concentration in the melt and its change during growth, crystallization mechanisms, and growth technology. To show that the phase diagram of the Li_2_O-Nb_2_O_5_-B_2_O_3_ system is favorable for the optical quality of the growing LiNbO_3_:B crystal, since crystallization proceeds from the two-phase region and the only crystallizing phase is LiNbO_3_.
- To show that concentrations of boron in the melt studied in the work provide a compositionally and optically homogeneous LiNbO_3_:B crystal with suppressed photorefraction, and up to 50% of the weight of the melt can be consumed. For example, when growing heavily doped LiNbO_3_:Zn crystals with suppressed photorefraction and high compositional and optical uniformity, no more than 20% of the melt is consumed.
- To show that the charge preparation method is not important for growing LiNbO_3_:B crystals with suppressed photorefraction. Photorefraction is completely suppressed in LiNbO_3_:B crystals grown from both homogeneously doped and directly doped charge.

The main goal of this work is to show that the growth of LN crystals from melts doped with boron is optimal. This method saves time and money. It provides a possibility to obtain large-sized compositionally and optically uniform, optically stable LiNbO_3_ crystals for laser radiation conversion. The optical and structural characteristics of LiNbO_3_:B crystals obtained from a charge of different genesis prove such a conclusion. The goal is also set by a detailed analysis of physicochemical particularities of crystallization in the Li_2_O-Nb_2_O_5_-B_2_O_3_ system. A much larger fraction of the melt crystallizes into a ready boule when growing LN from a boron-doped melt compared to the fraction of crystallized melt when growing LN crystals heavily doped with non-photorefractive metals (Mg, Zn, Gd, In, Sc, etc.).

## 2. Materials and Methods

Charge for LiNbO_3_:B crystal growth was obtained by two ways. Charge for sample 1 was obtained by homogeneous doping from a Nb_2_O_5_:B-Li_2_CO_3_ mixture; the Nb_2_O_5_:B precursor was used at this [20]. Boric acid H_3_BO_3_ was added directly into a hydrofluoric acid solution of niobium pentoxide, the preparation of which is described in detail in the work [20]. Charge for sample 2 was obtained by a direct solid-phase synthesis from a Li_2_CO_3_:Nb_2_O_5_:H_3_BO_3_ mixture [10]. The components of mixture were pure—not more than 10^−4^ impurities in each. Boron concentration in the Nb_2_O_5_:B precursor, charge and crystal samples 1 and 2 (cone and bottom parts of each crystals) was controlled at all synthesis and growth stages, Table 1.

All crystals investigated in this work were grown on the equipment Crystall-2 (Voroshilovgradsky zavod electronnogo mashinostroeniya, Voroshilovgrad, Russia). The crystal diameter was controlled by a special additional system incorporated into the installation.

**Figure 2:**
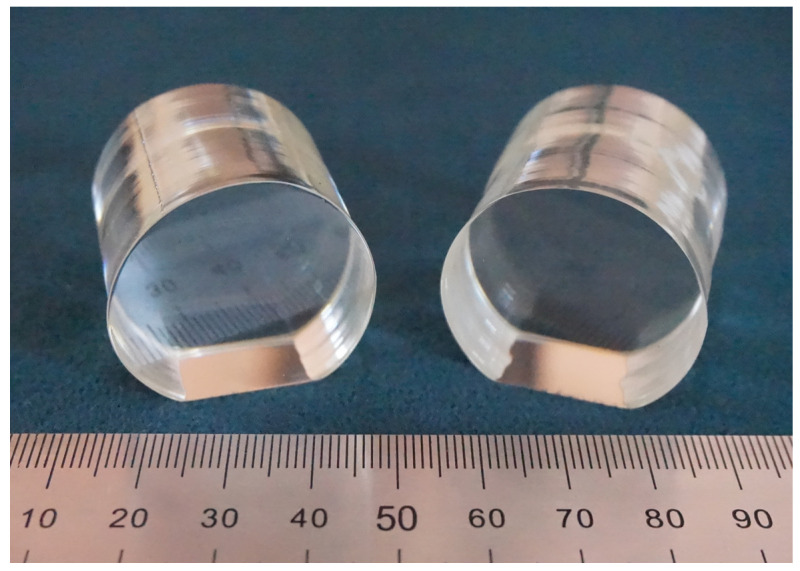
LiNbO_3_:B ((B) = 0.0034 and 0.0079 wt% in the charge) crystals; samples are turned to single domain state and polished.

Samples 1 and 2 were boules 28 to 30 mm in diameter and 40 mm long (cylindrical part), Figure 2. They were grown by Czochralski from a congruent melt. Crystals were grown in air in the (001) direction from platinum crucibles 75 mm in diameter. Growth rate was 0.61–0.63 mm/h, rotation rate was 14 rpm, and the axis gradient above the melt was 3 deg/cm. Sample 1 ((B) = 0.0034 wt% in the charge) was grown from a homogeneously doped charge; sample 2 ((B) = 0.0079 wt% in the charge) was grown from a directly doped charge. 

The technology of obtaining of granular charge with a high bulk density and growing of control crystals CLN and NSLN K_2_O are described in detail in paper [21].

Concentration of metal impurities in LiNbO_3_:B charge and crystals was determined by AES and MS-ICP methods of analysis, Table 2. AES (atomic emission spectroscopy) analysis was performed on spectrometer ICPE-9000 (Shimadzu, Japan, Kyoto). MS-ICP (mass spectrometry analysis with inductively coupled plasma) was performed using a quadrupole mass spectrometer ELAN 9000 DRC-e (PerkinElmer, Hopkinton, MA, USA).

CLN crystal was grown by Czochralski in air from platinum crucibles 75 mm in diameter under a relatively small (~4–6 deg/cm) axial gradient in the direction of the polar axis (z-cut) at speeds of rotation (~16–18 rpm) and displacement (~0.8 mm/h). The crystal growth rate was ~1.05–1.12 mm/h. Near-stoichiometric crystal NSLN K_2_O was grown due to a HTTSSG (High Temperature Top Seeded Solution Growth) technology from a congruent melt with the addition of 5.5 wt% K_2_O flux to the melt. The growth rate was low (<0.25 mm/h), and the after-growth annealing of the crystal boule was carried out at 1200 °C for 20 h.

All crystals, both studied and control, were turned to a single domain state by high temperature electrodiffusion annealing: a constant electrical voltage was applied to the polar cuts of the crystal during cooling at a rate of 20 deg/h in the temperature range 870–1230 °C.

To record PILS, samples were cut from the middle part of a cylindrical LN boule in the form of parallelepipeds 6 × 7 × 8 mm^3^ in size. The edges of the samples coincided in direction with the main crystallophysical axes. The faces of the parallelepipeds were carefully polished. Polished plates of Z-oriented LN crystals 1 mm thick were used to record conoscopic patterns. Experiments on PILS and conoscopy were carried out using the Nd:YAG laser MLL-100 (Changchun New Industries Optoelectronics Tech. Co. Ltd., Changchun, China), wavelength λ_0_ = 532 nm.

The integrated optical homogeneity of the LiNbO_3_:B and control crystals was studied by laser conoscopy. The transmission axes of the polarizer and analyzer were mutually orthogonal in the conoscopic experiments. The polarizer transmission axis made an angle of 45° with the vertical axis. The laser beam axis coincided with the optical axis of the crystal; the beam was normal to its input face. The samples were placed on a mobile x–y optical stage so that one can scan the entire input face by a laser beam and record a series of conoscopic patterns. The conoscopic patterns were excited by a laser beam power P = 90 mW.

In the PILS study, the laser radiation scattered by the crystal was incident on a semitransparent screen placed behind the crystal and recorded by a digital video camera. The scattering angle was measured by the formula θ = arctg(a/b), where θ is the PILS opening angle, a is the PILS indicatrix size along the positive Z direction, and b is the distance from the sample to the screen. The a size was measured by the PILS pattern point where scattered radiation decreases by one order of magnitude. In PILS experiments, the laser beam was directed along the y axis, and the electric field strength vector E of the laser radiation was parallel to the polar axis z of the crystal. The laser radiation intensity was I = 6.29 W/cm^2^. The obtaining details of PILS patterns were similar to the method described in [22].

XRD patterns of powdered LiNbO_3_:B crystal samples were determined by diffractometer DRON-6 (NPP Burevestnik, Sankt-Peterburg, Russian Federation). A pyrolytic graphite monochromator was placed in the primary beams (CuKα radiation, voltage 35 kV, current 20 mA). In the areas of reflections, XRD patterns were taken with more details with a step of 0.02°, and in the background areas, with a step of 0.2°. During the registration of an X-ray image, the stability of the registration scheme was monitored. The error of determining the intensity at each point of the diffraction line is at least 3%. XRD data on congruent and stoichiometric crystals are described in paper [23].

## 3. Results and Discussion

**Figure 3:**
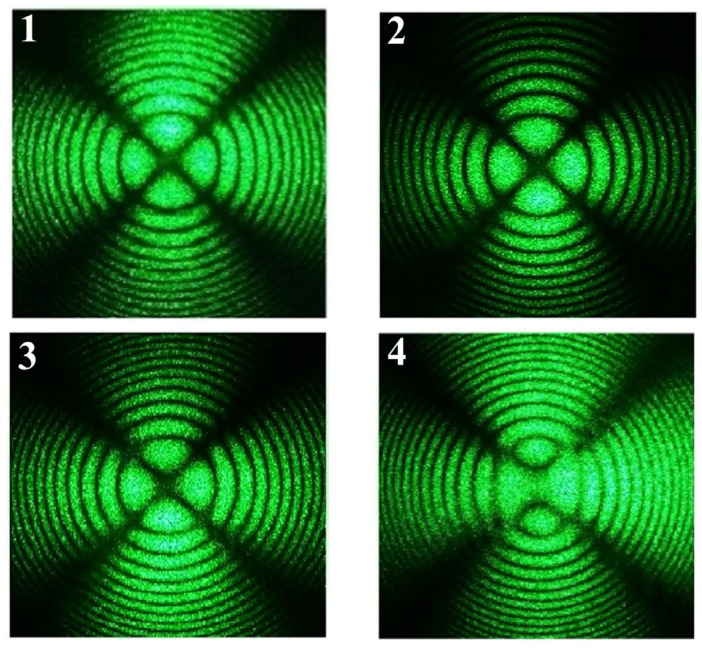
Conoscopic patterns of (**1**) sample 1, (**2**) sample 2, (**3**) CLN, (**4**) NSLN K_2_O.

Conoscopy investigates the optical uniformity of LN crystals. Figure 3 (1–3) demonstrates conoscopic patterns of samples 1, 2 and CLN. Conoscopic patterns of these three crystals have circular symmetry, the black Maltese cross remains intact in the center of the field of view, and the isochromes are concentric circles centered at the exit point of the optical axis. Such patterns are characteristic of uniaxial optically inactive crystal. There are no signs of anomalous optical biaxiality in any of the conoscopic patterns. These conoscopic patterns indicate the optical homogeneity of these LN samples and their good optical quality. The conoscopic pattern of NSLN K_2_O crystal (Figure 3 (4)) is much more defective. The conoscopic picture has pronounced signs of anomalous optical biaxiality: the Maltese cross is deformed in the vertical direction corresponding to the direction of deformation of the optical indicatrix of the crystal, with a break in the center of the cross; the isochromes are in the form of ellipses. All this testifies to the low optical homogeneity of the NSLN K_2_O crystal. Thus, samples 1 and 2 grown from a boron-doped melt have optical homogeneity at least as good as CLN crystal and noticeably better than NSLN K_2_O crystal.

**Figure 4:**
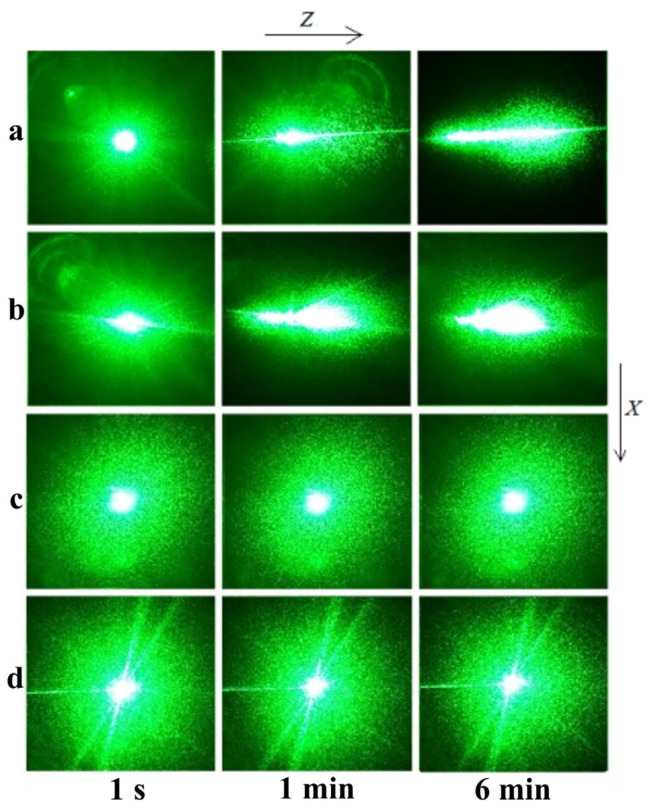
PILS patterns of (**a**) CLN, (**b**) NSLN K_2_O, (**c**) sample 1, (**d**) sample 2. I = 6.29 W/cm^2^.

PILS studies reveal optical damage resistance of the investigated LN crystals. Three-layer speckle structure of the CLN PILS pattern changes significantly over time, Figure 4a. The indicatrix from a round shape in the first second of PILS excitation transforms over time, first into an oval comet-like shape, then it takes the form of an asymmetric figure eight, the figure being oriented along the polar axis of the crystal. Over time, a larger “petal” of the figure eight develops in the positive direction of the polar axis, and a smaller one in the negative direction, Figure 4a. The opening angle of the CLN PILS indicatrix is 17°. All this indicates a relatively low but non-zero value of the photorefraction effect in the CLN crystal. The NSLN K_2_O crystal PILS indicatrix begins to open in the first seconds of irradiation; the opening angle is 29°. Thus, NSLN K_2_O photorefractive sensitivity is even higher than that of CLN. The PILS indicatrix opens in both CLN and NSLN K_2_O crystals and is accompanied by the destruction of the laser beam, Figure 4a,b. At the same time, the scattering pattern of LiNbO_3_:B samples 1 and 2 almost does not change with time, Figure 4c,d. Even at a radiation power of 6.29 W/cm^2^, there is no photorefractive response, the PILS indicatrix is not revealed, and only circular scattering of the laser beam by static structural defects is observed, Figure 4c,d. The scattering angle is no more than 3 degrees. The scattering pattern retains a shape close to a circle throughout the experiment (6 min), Figure 4c,d. Such temporal behavior of PILS patterns, absence of the PILS indicatrix and the absence of laser beam destruction indicate suppression of the photorefraction effect in LiNbO_3_:B samples 1 and 2 crystals.

PILS studies have revealed that nominally pure control crystals CLN and NSLN K_2_O have a strong photorefractive response. Contrary to them, photorefractive sensitivity in LiNbO_3_:B crystals is suppressed, Figure 4. At this, B concentration in grown crystals is at traces level, 10^−4^–10^−5^ wt%. 

This optical behavior of LiNbO_3_:B crystals is similar to the behavior of strongly doped LiNbO_3_:Zn and LiNbO_3_:Mg crystals. Photorefractive effect is suppressed in LiNbO_3_:Zn and LiNbO_3_:Mg crystals doped with near- or after-threshold concentrations [24,25,26,27,28,29]. Only round scattering of laser beam on static structure defects is observed in both LiNbO_3_:B and LiNbO_3_:Zn, LiNbO_3_:Mg crystals. The PILS indicatrix does not open; the laser beam is not distracted.

Such features of the optical properties of LiNbO_3_:B crystals are due to the physicochemical characteristics and structure of the boron-containing melt; they are determined by the crystallization mechanisms and growth technology. For example, the structure of LN melt containing boron is highly sensitive towards boron concentration; it cannot contain more than 0.18 wt% boron. Otherwise, a viscous film forms on the melt surface; the film prevents seeding; crystal grown from such a melt has many micro- and macro-defects unremovable by post-growth treatment. Growing of LiNbO_3_:B crystals from melts doped with boron required new decisions and adaptation of the commonly used technology for the growth of nominally pure and metal-doped LN crystals. This affected not only the technological parameters of growth and the internal equipment of the growth chamber, but also the methods of synthesis of the initial charge [10,11]. Boron, being an LN dopant, changes the melt structure; thus, it changes the crystal structure and properties but does not incorporate into LiNbO_3_:B crystal, Table 1.

**Figure 5:**
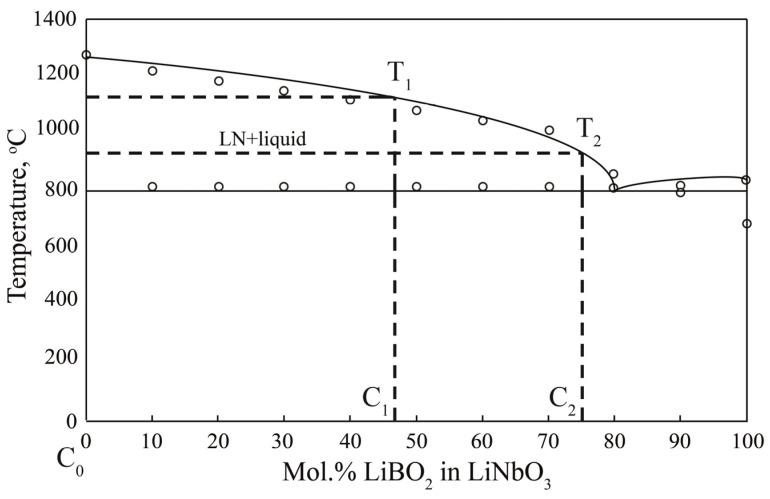
Polythermal incision LiNbO_3_-LiBO_2_ of a quasi-ternary Li_2_O-Nb_2_O_5_-B_2_O_3_ system state diagram. C_0_, C_1_ and C_2_ are the melt composition or boron concentration in the melt; T_1_ and T_2_ are the crystallization temperatures corresponding to melt compositions C_1_ and C_2_. The figure is reproduced from the paper *Crystals* **2021** *11(5)* 458 10.3390/cryst11050458 (ref. [9]).

Due to polythermal incision, LiNbO_3_-LiBO_2_ of a quasi-ternary Li_2_O-Nb_2_O_5_-B_2_O_3_ system state diagram boron thermodynamically cannot incorporate into the LiNbO_3_:B crystal; the LN phase in a solid state has no solubility area for boron and its compounds. The polythermal incision LiNbO_3_-LiBO_2_ of a quasi-ternary Li_2_O-Nb_2_O_5_-B_2_O_3_ system state diagram is presented on Figure 5 and in [5,9]. This fact explains an extremely low distribution coefficient K_D_ for boron in LN crystal. The incision has a relatively simple form, since it contains only one two-phase eutectic transformation and does not have solubility regions. C_0_, C_1_ and C_2_ in Figure 5 and in [9] are the melt composition or boron concentration in the melt, T_1_ and T_2_ crystallization temperature corresponding to the melt composition C_1_ and C_2_.

Such a phase diagram type is favorable for the growing LiNbO_3_:B crystal quality, since crystallization originates from a two-phase region and the only crystallizing phase is LiNbO_3_. If we could consider only the phase diagram and not take into account melt changes at doping by boron, then the crystal grown from such a melt should be chemically and structurally equivalent to a congruent undoped crystal grown from a congruent melt. Yet, in reality, the LiNbO_3_:B crystal has a Nb_Li_ defects concentration close to that of a stoichiometric crystal. The real LiNbO_3_:B has a higher structure order than a CLN crystal [11]. The reason probably lies in a boron electron structure; the element is a strong complexing agent due to a single electron at *p* orbital. This provides high ionization energies and electronegativity values at a small ionic radius and leads to a significant change in the structure of the LN melt when boron is added.

Studies of the influence of the melt structure on the crystallization processes are highly relevant in modern science. The main concept considers a melt as a set of clusters of different electrochemical activity and structure [30]. Lack of equipment and experimental difficulties did not allow us to study how the Li_2_O-Nb_2_O_5_-B_2_O_3_ system melts composition influences the type and electrochemical activity of the complexes in the melt. This is why analysis and comparison of grown LiNbO_3_ crystals are relevant as a possibility to interpret melt properties (electrochemical activity of ionic complexes, the role of dopant, the value of stoichiometry, etc.). The study should analyze and compare properties of LN crystals.

Strictly speaking, when any dopant is introduced into a congruent LN melt, it ceases to be congruent. This, in particular, means that the spectrum of variations of ionic complexes in the melt in terms of structure and components greatly increases [31]. The capture of the melt during crystallization occurs in a limited zone near the crystallization front, where temperature is constant. Thus, the only complexes that will crystallize are those for which the temperature is solidus (T_C1_), of course, considering some overcooling [31]. As some melt volume is used, concentrations of other ion complexes in the rest melt part change. The fraction of complexes with the T_C1_ solidus temperature will decrease. At some critical point, there will be not enough T_C1_ complexes to continue crystallization with a constant distribution of dopant along the crystal polar axis in a crystal. This fact limits the fraction of the melt that can be crystallized into a compositionally uniform doped LN crystal. This limitation is true for any LN dopant known so far. After the system reaches the abovementioned critical point, its behavior can be different: from a significant change in the dopant concentration to such defects as cellular growth and crystallization of a phase of a different composition and structure [32].

A lot of factors influence the condition of achievement of this critical point; they influence such parameters as possible dimensions of an optically homogeneous crystal and the constancy of the dopant concentration throughout the volume of the crystal boule. The factors are the composition of the melt, the thermodynamics of the initial components, the graphic expression of the phase diagram, the structure of the melt, consisting of ionic complexes with different thermodynamic and kinetic characteristics, and even the technical capabilities (sensitivity and reaction time constant) of the growth process control and monitoring system. An important conclusion follows from this discussion for the technology of doped LN crystals: only when a certain part of the melt crystallizes is it possible to grow doped LN crystals with a uniform dopant distribution over the volume by the Czochralski. Consequently, the length and diameter of the crystal boule of such a crystal is limited. In different systems and at different dopant concentrations, the limiting sizes of such structurally and compositionally uniform LiNbO_3_ crystals differ. Different physical and chemical causes can serve as a reason for this. The causes manifest as differences in the thermodynamic parameters of systems, that is, in the phase diagrams type. For example, in case of LiNbO_3_:Zn, crystals grow compositionally and optically uniform only when 20% of the melt crystallizes [24].

LN has a homogeneity area neither with boron, nor with its compounds in a boron-containing system Li_2_O-Nb_2_O_5_-B_2_O_3_. Thus, in such a system, the only crystallizing phase is LiNbO_3_, Figure 5. Boron concentration in the melt increases during crystallization; thus, the crystallization temperature decreases and the viscosity of the melt increases. The latter limits convection flows in the melt, Figure 5. These facts probably limit the maximal possible boron concentration in the LN melt to 0.18 wt%. Exceeding this threshold leads to cellular growth and other fatal defects (for example, in the form of “channels”) in a LiNbO_3_:B crystal [10]. In our work, boron concentration in the melt is limited to (B) ≤ 0.008 wt%. On the one hand, boron in this concentration still influences the melt structure and LiNbO_3_:B crystal properties. On the other hand, such a concentration is small enough and allows us to grow compositionally and optically uniform crystals with crystallization of up to 50% melt.

**Figure 6:**
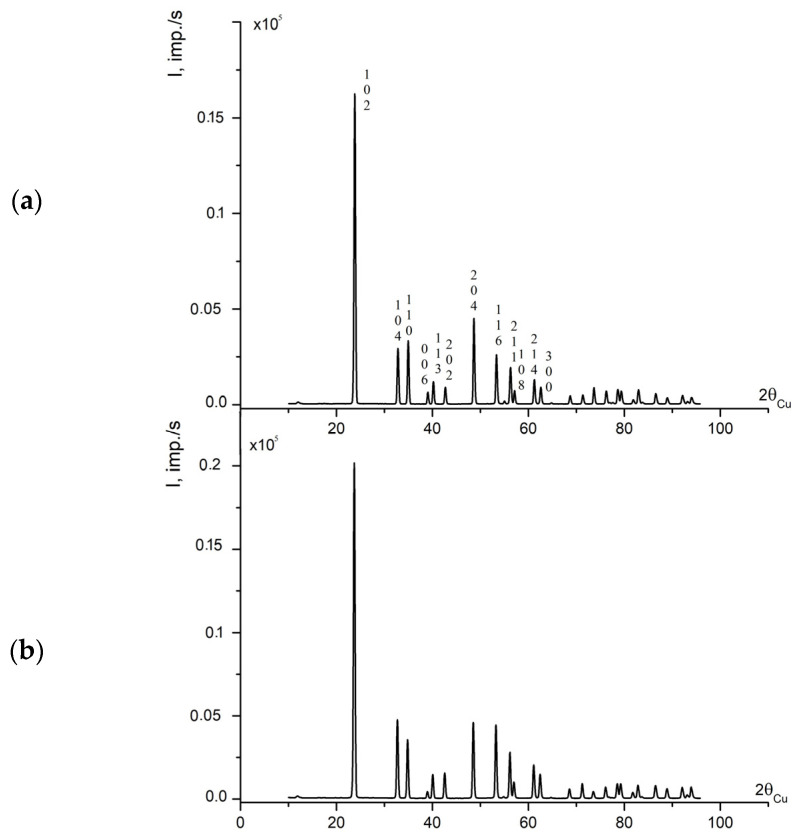
X-ray diffraction patterns of powdered sample 2 (**a**) and sample 1 (**b**).

Figure 6a,b demonstrates XRD patterns of powder samples of the studied samples 1 and 2. XRD patterns of samples are alike and correspond to patterns of LN with a symmetry space group *R*3*c* with two formula units in a unit cell [15]. Table 3 demonstrates refinement of unit cell period and structure characteristics (atom coordinates and population factors) of samples 1 and 2, and control crystals CLN and NSLN K_2_O [23,33]. Structure characteristics of samples 1 and 2 (atom coordinates and population factors *G*) characterize cations distribution in oxygen octahedra O_6_; the data are given in Table 4.

Table 3 shows that unit cell parameters of samples 1 and 2 are close to that of NSLN K_2_O. This confirms earlier conclusions made on the basis of Raman spectroscopy in polarized radiation and IR spectroscopy studies carried out for LiNbO_3_:B crystals of different compositions [9,12,14]. Both present and earlier data indirectly indicate an increase in stoichiometry (R = (Li)/(Nb)) at LN doping with traces of boron. 

Data from Table 4 indicate that despite the doping type, LiNbO_3_:B crystals have such point defects as niobium cations located in lithium (Nb_Li_) and vacant (Nb_V_) oxygen octahedra O_6_ of a perfect stoichiometric LN structure. The technique for analyzing the defect structure using full-profile XRD analysis (the Rietveld method) and the general logic of reasoning are described in the works [22,23,33].

Note that Nb_Li_ defects are deep electron traps; they substantially influence photorefractive and luminescent LN properties. At this, concentrations of Nb_Li_ and Nb_V_ defects are almost equal in sample 1 and 2. Population factors of lithium (G_Li_) and niobium (G_Nb_) sites are almost the same in sample 1 and are equal to 0.98 and 0.97, respectively. At the same time, G_Li_ and G_Nb_ values are more different from each other in sample 2 and are equal to 0.99 and 0.93, respectively. The value R = (Li)/(Nb) in sample 1 is 1.01 and in sample 2 it is 1.06. In both samples, the concentration of lithium atoms in lithium octahedra is higher than the concentration of niobium atoms in niobium octahedra of a perfect stoichiometric structure. Thus, boron as a dopant increases LN crystal stoichiometry. 

At the same time, the population factors ratio in sample 1 is closer to that of NSLN K_2_O than sample 2, Table 4. Table 4 also demonstrates that niobium ions’ incorporation to vacant LN structure octahedra changes a perfect alteration of intrinsic (Li, Nb) cation and vacancies along the polar axis, which disorders the cation sublattice of the LN structure.

**Figure 7:**
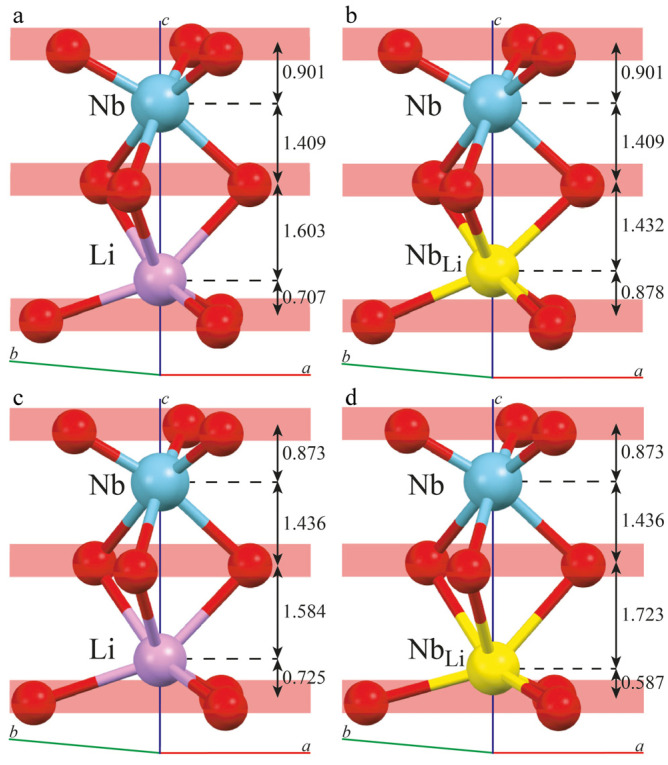
Location of cations in oxygen–octahedra clusters MeO_6_ in oxygen planes in crystals: (**a**,**b**)—sample 1; (**c**,**d**)—sample 2.

Metal–oxygen (Me-O) distances in MeO_6_ clusters and the metal–metal (Me-Me) distances along the polar axis were calculated from refined atom coordinates and unit cell parameters (Figure 7, Table 5).

Long and short Me-O distances of main motives (LiO_6_ and NbO_6_) of sample 1 are in a good correlation with the corresponding values of NSLN K_2_O crystal, Table 5. The Me-O bonds of sample 2 are significantly different; this proves a great distortion of its oxygen octahedra, Table 5. The most notable difference in Me-O distance is characteristic of the LiO_6_ clusters of sample 2: long distances increase at 0.025 Å; short, at 0.1 Å compared to the distances in a NSLN K_2_O crystal, Table 5. Despite this fact, the difference between short and long distances in the LiO_6_ clusters in sample 2 is the same (0.1 Å) as in the NSLN K_2_O crystal cluster. At the same time, at niobium incorporation to a lithium site in sample 1, there are no notable changes in corresponding distances of LiO_6_ clusters of a main motive. Incorporation of niobium to a vacant lithium site causes greater changes in distances along the polar axis in sample 1 than in sample 2, Table 5. It should also be noted that niobium incorporation to a lithium site causes shortening of short and extension of long distances in the LiO_6_ clusters of sample 2. 

Figure 7 demonstrates the location of intrinsic cations (Li and Nb) in main motive and near point anti-site defects Nb_Li_. The location is shown in oxygen octahedral clusters MeO_6_ of both studied LiNbO_3_:B crystals relative to the planes of oxygen atoms. Point defect Nb_Li_ in sample 1 is shifted to the octahedron center along the polar axis from the bottom oxygen plane; this should decrease its spontaneous polarization, Figure 7. The situation in sample 2 is opposite: the defect Nb_Li_ is shifted from the center towards the bottom oxygen plane, which should vice versa increase spontaneous polarization and Curie temperature. Niobium position in both LiNbO_3_:B samples is the same as in NSLN K_2_O. The found structural features explain the experimentally established increase in Curie temperature in LiNbO_3_:B crystals compared to the nominally pure CLN crystal discussed in papers [9,10,11].

The structure of LiNbO_3_:B crystals is nearing that of NSLN K_2_O crystals considering the complexing action of boron on the LN melt of congruent composition. Optical damage is substantially decreased and optical homogeneity is increased at this, due to a decrease in the amount of Nb_Li_ defects, anti-site defects acting as deep electron traps. The crystals’ purity can also increase as the result of the melt cleaning from different impurities when boron forms different complexes with them in a Li_2_O-Nb_2_O_5_-B_2_O_3_ system. This leads to an increase in optical quality: optical uniformity and optical damage resistance. Boron forms complexes with impurity metal cations which, thus, do not incorporate into the crystal and stay in the melt. 

In order to prove such a possibility, we have ab initio calculated Gibbs energies (isobaric–isothermal potential) of a hypothetically possible row of impurity metals borates (Al_4_B_2_O_9_, CaB_2_O_4_, CaB_4_O_7_, Ca_2_B_2_O_5_, Ca_3_B_2_O_6_, PbB_2_O_4_). The impurities might be present in the melt of the Li_2_O-Nb_2_O_5_-B_2_O_3_ system, Table 6.

In order to calculate changes in the isobaric–isothermal potential with increasing the temperature correctly, we have applied true heat capacity equations. Energy changes are associated with phase transitions of each of the participants in the reaction. The energy changes are considered in the temperature ranges applicable for the mentioned equations. True heat capacity equations correspond to an infinitesimal increase in heat with an infinitesimal change in temperature. The equation allows one to transit from thermodynamic functions at one temperature (298.15 K) to their values at another, which is within the range of applicability of the heat capacity equation.

We consider isobaric–isothermal potential as a change in the internal energy of the system during the implementation of chemical interaction. It allows one to evaluate the hypothetical possibility of a chemical reaction. A chemical reaction spontaneously proceeds in the direction of the formation of reaction products (G < 0) at p, T = const; at G = 0, chemical equilibrium is established; at G > 0, the chemical equilibrium shifts towards the initial reagents.

Impurity metals cations (Al, Ca, Pb, etc.) are inevitable in congruent charge at trace amounts. They transfer to the growing crystals and influence LN characteristics important for their future applications. Such impurities color LN crystals, increase the photorefractive effect and lead to the appearance of scattering centers. The authors of [34] were the first to bind some amount of Al^3+^ cations by the formation of poorly soluble aluminum borate Al_5_BO_9_. This decreased the concentration of Al_2_O_3_ in the reacting mixture. Based on calculations, we have assumed a ‘cleaning’ action of B_2_O_3_ flux on other impurity ions in the LN melt.

Available data on thermodynamic values [35,36,37,38,39,40] were used to calculate Gibbs energies of the following hypothetically possible chemical reactions in the Li_2_O-Nb_2_O_5_-B_2_O_3_ system:

2Al_2_O_3_ + B_2_O_3_ = Al_4_B_2_O_9_

CaO + B_2_O_3_ = CaB_2_O_4_

CaO + 2B_2_O_3_ = CaB_4_O_7_

2CaO + B_2_O_3_ = Ca_2_B_2_O_5_

3CaO + B_2_O_3_ = Ca_3_B_2_O_6_

PbO + B_2_O_3_ = PbB_2_O_4_

Concentrations of a number of regulated elements (Co, Mo, Si, Fe) do not decrease in samples 1 and 2. This is probably due to the insufficient concentration of boron cations for their binding in the boron-containing LN melt. Despite other elements, these ones do not form strong complexes with boron and thus, transit into the melt. However, this does not spoil the optical properties and composition of grown crystals. Concentrations of Pb, Ni, Cr, Ti, Al, Te, Sb and Bi cations are lower in samples 1 and 2 than in their charge (Table 2). The strongest decrease is observed for Al and Bi in sample 1; the concentration in crystal is 3 × 10^−4^ wt% smaller than that in the charge. This is in a good agreement with the change in the isobaric–isothermal potential calculated by us for the formation of the Al_4_B_2_O_9_ compound (−86.528 kJ/mol), Table 6. The calculated value of the change in the isobaric–isothermal potential of PbB_2_O_4_ compound formation (−39.873 kJ/mol) confirms the experimentally found decrease in the Pb cation concentration at 1 × 10^−4^ wt% in samples 1 and 2 compared to their charges, Table 2. The Ca ions concentration decrease in sample 2 is in a good agreement with the calculated change in the isobaric–isothermal potential of a number of compounds, Table 6: CaB_2_O_4_ (−112.586 kJ/mol), CaB_4_O_7_ (−116.118 kJ/mol), Ca_2_B_2_O_5_ (−181.428 kJ/mol), Ca_3_B_2_O_6_ (−234.669 kJ/mol). However, the Ca ions concentration does not decrease in sample 1. This may be due to the lower concentration of boron cations in the charge obtained by homogeneous doping (0.0034 wt%) than in the charge obtained by direct solid-phase doping (0.0079 wt%), Table 1.

Data (Table 6) show that the Gibbs energy change for all studied chemical reactions is negative. This only confirms our earlier assumption about the possibility of impurity metals (Al, Ca, Pb) binding by borates in a congruent melt. Table 2 data also confirm this conclusion. For the majority of metallic impurities, their concentration in the crystal is less than in the initial charge, Table 2.

## 4. Conclusions

LiNbO_3_:B crystals were grown by Czochralski from a charge of different genesis. Nominally pure CLN and NSLN K_2_O served as control.

LiNbO_3_:B crystals of different genesis were shown to have an optical uniformity not less than CLN and much more than NSLN K_2_O. At this, LiNbO_3_:B crystals have a significantly smaller photorefractive sensitivity than CLN and NSLN K_2_O crystals.

The crystallization from the Li_2_O-Nb_2_O_5_-B_2_O_3_ system is favorable for optically uniform crystals. Analysis of physicochemical particularities of crystallization in the Li_2_O-Nb_2_O_5_-B_2_O_3_ system revealed that a much larger fraction of the melt (>50%) crystallizes into a ready boule when growing LN from a boron-doped melt compared to the fraction of crystallized melt (<20%) when growing LN crystals heavily doped with non-photorefractive metals (Mg, Zn, Gd, In, Sc etc.)

XRD studies revealed a smaller Nb_Li_ concentration in LiNbO_3_:B crystals than in CLN crystals. Their concentration in LiNbO_3_:B is also close to that in NSLN K_2_O. At this, sample 1 is closer in composition to NSLN K_2_O than sample 2.

XRD confirms the localization of B cation in the face of a tetrahedral O_4_ void in LN structure. 

The LiNbO_3_:B crystals are purified when boron forms strong compounds with impurity metal cations in the Li_2_O-Nb_2_O_5_-B_2_O_3_ system. Impurities are thus removed from the crystallization process.

## Figures and Tables

**Table 1:** Boron concentration in Nb_2_O_5_:B precursor, initial charge and LiNbO_3_:B crystals obtained due to different technologies.

		Boron Concentration, wt%	
		Sample 1	Sample 2
In Nb_2_O_5_:B precursor		0.0042	-
In charge		0.0034	0.0079
In crystal	Cone	8 × 10^−5^	3.2 × 10^−4^
	Bottom	9 × 10^−5^	3.6 × 10^−4^

**Table 2:** Impurity composition of charge and samples 1 and 2 crystals.

Impurity	Concentration			
	In Homogeneously Doped Charge, wt%	In Directly Doped Charge, wt%	In Sample 1, wt%	In Sample 2, wt%
Mn, V, Mg, Sn	<3 × 10^−4^	<3 × 10^−4^	<2 × 10^−4^	<2 × 10^−4^
Pb, Ni, Cr	<5 × 10^−4^	<4 × 10^−4^	<4 × 10^−4^	<3 × 10^−4^
Co, Mo	<2 × 10^−4^	<3 × 10^−4^	<2 × 10^−4^	<3 × 10^−4^
Si, Fe	<3 × 10^−4^	<4 × 10^−4^	<3 × 10^−4^	<4 × 10^−4^
Ti	<4 × 10^−4^	<3 × 10^−4^	<2 × 10^−4^	<2 × 10^−4^
Al	<7 × 10^−4^	<6 × 10^−4^	<4 × 10^−4^	<5 × 10^−4^
Zr	<3 × 10^−4^	<5 × 10^−4^	<4 × 10^−3^	<3 × 10^−3^
Ca	<5 × 10^−4^	<6 × 10^−4^	<5 × 10^−4^	<4 × 10^−4^
Te, Sb	<4 × 10^−4^	<5 × 10^−4^	<3 × 10^−4^	<4 × 10^−4^
Bi	<4 × 10^−4^	<3 × 10^−4^	<1 × 10^−4^	<2 × 10^−4^

**Table 3:** Unit cell parameters of NSLN K_2_O, CLN and samples 1 and 2.

Unit Cell Parameters	NSLN K_2_O	CLN	Sample 1	Sample 2
*a*, Å	5.1429	5.1483	5.1476	5.1450
*c*, Å	13.8447	13.8631	13.8594	13.8561

**Table 4:** Refined atoms coordinates (x/a, y/b, z/c) and population factors G in samples 1 and 2.

	G	x/a	y/b	z/c		G	x/a	y/b	z/c
Sample 1 (R_wp_ (%) = 10, R_p_ (%) = 7.63)					Sample 2 (R_wp_ (%) = 12.39, R_p_ (%) = 9.07)				
Nb	0.97	0	0	0	Nb	0.93	0	0	0
O	1.00	0.0656	0.3393	0.0653	O	1.00	0.0805	0.324	0.063
Li	0.98	0	0	0.2827	Li	0.99	0	0	0.282
Nb_Li_	0.016	0	0	0.2950	Nb_Li_	0.018	0	0	0.272
Nb_V_	0.009	0	0	0.135	Nb_V_	0.01	0	0	0.135

**Table 5:** Interatomic distances in NSLN K_2_O and studied LiNbO_3_:B crystals.

Atom Pairs	NSLN K_2_O	Sample 1	Sample 2
Nb-O distances in NbO_6_ cluster of a main motive (Å)			
Nb-O	2.096	2.099	2.138
Nb-O	1.842	1.839	1.738
Li-O distances in LiO_6_ cluster of a main motive (Å)			
Li-O	2.247	2.233	2.272
Li-O	2.142	2.158	2.240
Nb_Li_-O distances in Nb_Li_O_6_ cluster (Å)			
Nb_Li_-O	2.267(3)	2.220	2.340
Nb_Li_-O	2.133(1)	2.114	2.232

**Table 6:** Gibbs energy calculated for chemical reactions in a congruent LN melt with a B_2_O_3_ flux.

№	Compound	ΔG, kJ/mol	T, K
1	Al_4_B_2_O_9_	−86.528	1308
2	CaB_2_O_4_	−112.586	1573
3	CaB_4_O_7_	−116.118	1573
4	Ca_2_B_2_O_5_	−181.428	1573
5	Ca_3_B_2_O_6_	−234.669	1573
6	PbB_2_O_4_	−39.873	1400

## Data Availability

The raw data required to reproduce these findings are available from corresponding author D.M. on a reasonable request.

## Abbreviations

LN	lithium niobate, LiNbO_3_
CLN	congruent lithium niobate
PILS	photoinduced light scattering
NSLN K_2_O	near-stoichiometric lithium niobate crystal obtained from a congruent melt with addition of K_2_O flux
HTTSSG	high temperature top seeded solution growth
XRD	X-ray diffraction
